# Treatment with IL5-/IL-5 receptor antagonists in drug reaction with eosinophilia and systemic symptoms (DRESS)

**DOI:** 10.1007/s40629-022-00224-7

**Published:** 2022-08-23

**Authors:** Anna Gschwend, Arthur Helbling, Laurence Feldmeyer, Ulrich Mani-Weber, Cordula Meincke, Kristine Heidemeyer, Simon Bossart, Lukas Jörg

**Affiliations:** 1 Division of Allergology and Clinical Immunology, Department of Pneumology and Allergology, Inselspital, Bern University Hospital, University of Bern, Bern, Switzerland; 2 Department of Dermatology, Inselspital, Bern University Hospital, University of Bern, Bern, Switzerland; 3 Allergiepraxis Beo, Thun, Switzerland

**Keywords:** Eosinophilia, Benralizumab, Mepolizumab, Reslizumab, Drug hypersensitivity

## Abstract

**Purpose:**

Drug reaction with eosinophilia and systemic symptoms (DRESS) is a severe delayed drug hypersensitivity reaction with exanthema, eosinophilia, and organ manifestations. After culprit drug withdrawal, systemic corticosteroids (CS) are the most widely used treatment, often requiring high doses for months. Blocking the IL-5/IL‑5 receptor axis with mepolizumab, reslizumab, and benralizumab is a promising targeted treatment with a good safety profile and no immunosuppressive effect. The aim of this study is to summarize current experience with the anti-IL5/IL-5-receptor therapy in DRESS.

**Methods:**

A retrospective analysis of all patients diagnosed with DRESS and treated with mepolizumab, reslizumab, or benralizumab in DRESS was performed. In addition, a PubMed–Medline search for publications on DRESS with anti-IL-5/IL‑5 receptor treatment was performed.

**Results:**

Of the 14 cases identified, 6 patients were treated with mepolizumab, 6 with benralizumab, 1 patient with reslizumab, and 1 patient was switched from benralizumab to mepolizumab. The main indication for an IL‑5 blockade was a therapy-refractory course (7/14 [50.0%]), recurrent relapses (3/14 [21.4%]), and severe organ dysfunction (2/14 [14.3%]). In 13/14 (93%) cases, a rapid clinical improvement with suppression of eosinophilia and reduction of CS could be achieved. In all but two cases under mepolizumab (dose 100–600 mg) or reslizumab (dose according to body weight), two or more doses were necessary until resolution of DRESS. In 4/7 cases under benralizumab, a single 30 mg dose was sufficient.

**Conclusion:**

Blockade of the IL-5/IL‑5 receptor axis appears to be a promising treatment in DRESS with fast clinical improvement, which may allow more rapid reduction of CS, and a good safety profile. In addition, a summary of recommendations on when to use blockade of the IL-5/IL‑5 receptor axis in DRESS treatment is provided.

## Introduction

Drug reaction with eosinophilia and systemic symptoms (DRESS) is a rare but severe, delayed drug hypersensitivity reaction (DHR) [1]. DRESS can mimic infections, hematologic malignancy, and autoimmune diseases with the main feature of (often) severe eosinophilia [2]. Organ manifestations involving kidneys, gastrointestinal tract, lungs, heart, and liver are typical, with the liver being the most commonly affected organ [3]. The course of DRESS is frequently characterized by viral reactivations such as human herpesvirus 6 (HHV6), HHV7, Epstein-Barr virus (EBV), and cytomegalovirus (CMV) [3, 4]. Recurrent spontaneous relapses occurring for months, triggered by viral reactivation, reduction of systemic corticosteroids (CS) or immunosuppressive drugs, or introduction of new drugs, are typical in the DRESS disease course [5]. Complications of DRESS—even after years—include the development of autoimmune diseases, multiple drug hypersensitivity, or persistent organ damage [5, 6]. Even under careful treatment, mortality from DRESS ranges from 2 to 10% [3].

Treatment of DRESS is very challenging. The most important measure is the removal of the causative drug. In many situations the use of immunosuppressive drugs is vital [1, 7]. Data on DRESS treatments are mainly based on retrospective analyses and are not standardized. CS are the most widely used drugs, often requiring high doses for weeks up to months [1, 7]. For refractory cases or those with frequent relapses, additional immunosuppressive drugs such as cyclosporine, cyclophosphamide, and Janus kinase (JAK) inhibitors are used [8–10]. However, long-time immunosuppression with CS coincides with substantial side effects; moreover, opportunistic infections may complicate the disease [6]. Therefore, targeted treatment without a significant immunosuppressive effect and a lower side effect profile could be very beneficial in the treatment of DRESS. Since DRESS is driven by IL‑5, the blockage of the IL‑5 axis would be an ideal target [11]. IL‑5 leads to the expansion of eosinophil granulocytes and prevents their apoptosis [12]. Blockage of the IL-5/IL-5R axis is promising and has been well studied in recent years within numerous diseases such as eosinophilic asthma [13], eosinophilic granulomatosis with polyangiitis (EGPA) [14], chronic rhinosinusitis with polyposis [15], hypereosinophilic syndrome [16], and eosinophilic esophagitis [17, 18]. While two of the humanized monoclonal antibodies, mepolizumab and reslizumab, target the alpha chain of IL‑5, benralizumab targets the alpha subunit of the IL‑5 receptor (IL-5R). All three drugs have a good safety profile without significant immunosuppression which is promising for potential and optimal steroid-sparing agents in DRESS [19–21]. To date, only single case reports of treatment success with anti-IL‑5 drugs in DRESS have been published [22–29]. The aim of this study is to report our experience with anti-IL5 therapy in DRESS and to summarize the current literature.

## Methods

A retrospective analysis of all patients diagnosed with DRESS and treated with mepolizumab, reslizumab, or benralizumab in DRESS was performed. Cases were obtained from a drug allergy database of the Division of Allergy and Clinical Immunology, Inselspital Bern. The study was approved by the local ethics committee (Kantonale Ethikkommission Universität Bern ID 2018-02192), all patients signed the informed consent. Medical records of each patient were reviewed for clinical features such as exanthema, fever, eosinophilia, presence of atypical lymphocytes, organ involvement, involved drugs, dose and duration of IL-5/IL-5R blockade, and outcome. We calculated the Regiscar score for each case based on clinical features, which classifies a DRESS as definitive case (≥ 6 points), probable case (4–5 points), possible case (2–3 points), or no case (< 2 points) [3]. To supplement our data with published cases, we searched PubMed–Medline for publications on DRESS with IL-5/IL-5R treatment with the following terms: drug reaction with eosinophilia and systemic symptoms, DRESS, severe drug hypersensitivity, drug-induced hypersensitivity syndrome or DIHS and IL‑5 blockade, IL‑5 treatment, benralizumab, mepolizumab, reslizumab. The literature was evaluated for clinical features of DRESS, indication for IL‑5 blockade, dose and duration of IL-5/IL-5R blockade, and outcome. Study analysis was performed using Graphpad Prism 8 (GraphPad Software, Inc, La Jolla, CA, USA). All results are summarized with descriptive statistics. Proportions are expressed as percentages.

## Results

In our database, we identified 4 patients with a DRESS diagnosis, based on clinical signs with a Regiscar score of > 4 points and treated with anti-IL5/IL-5R antibodies [3]. Three individuals were included in our study, one refused to participate (Table 1). The PubMed–Medline search revealed 11 cases published with DRESS and treated with anti-IL5 drugs (Table 2; [22–29]). Of the 14 cases, 6 were treated with mepolizumab, 6 with benralizumab, 1 with reslizumab, and 1 was switched from benralizumab to mepolizumab during the DRESS disease course. The median age was 58 years (interquartile range [IQR] 49.5–68.5), the proportion of females was 8/14 (57.1%). Indication for an IL‑5 blockade was a therapy-refractory course under CS with persisting or increasing eosinophilia (6/14 [42.9%], cases 1, 4–5, 7–9), severe organ dysfunction (3/14 [21.4%], cases 3, 6, 12), recurrent relapses (3/14 [21.4%], cases 1, 11, 13), continued treatment with the causing drug (1/14 [7.1%], case 14), and concomitant septic shock (1/14 [7.1%], case 2). The median Regiscar score was 6 [3]. All patients were initially treated with high dose systemic steroids (> 50 mg prednisone equivalent per day); two with additional immunosuppressive drugs (cases 10 and 11), three with intravenous immunoglobulins (cases 2, 6, and 13).

**Table 1:** Patient characteristics of three cases with drug reaction with eosinophilia and systemic symptoms (DRESS) from the Inselspital Bern database

	Patient 1	Patient 2	Patient 3
*Demographics*			
Age	62	70	39
Gender	m	m	m
*Culprit drug*	Amoxicillin	Piperacillin Vancomycin Meropenem	Metamizol suspected
*Organ involvement*			
Regiscar score	7	6	6
Eosinophilia (max.)	4.75 G/l	4.16 G/l	1.28 G/l
Lymphadenopathy	Yes	No	Yes
Fever	Yes	Yes	Yes
Exanthema	Yes	Yes	Yes
Features of exanthema	Generalized MPE Skin desquamation	Generalized erythema with pinpoint pustules Skin blisters and erosion	Generalized MPE Skin desquamation
Liver	Yes	No	Yes
Kidney	Yes	Yes	No
Lung	No	No	No
Heart	No	No	Yes
Viral reactivation	No	n/a	No
*Basic treatment*	Methylprednisolone/Prednisolone	Solucortef Prednisolone IVIG	Prednisolone
*IL-5/IL-5R antagonist used*	Mepolizumab	Mepolizumab	Benralizumab
Indication	Therapy refractory under systemic steroids	Severe sepsis under systemic steroids	Persisting hepatitis under systemic steroids
Number of doses	3	1	3
Outcome	Rapid improvement, systemic steroid tapering possible	Minor improvement	Improvement of hepatitis, steroid tapering possible

*IVIG* intravenous immunoglobulin, *MPE* Maculopapular exanthema, *M* male, *n/a* not applicable, *G/L* Giga per litre

**Table 2:** Published cases on drug reaction with eosinophilia and systemic symptoms (DRESS) and IL-5/IL-5R blockade

Case	Gender	Age	Reaction	Regiscar	Trigger	Max. Eos G/l	IL‑5 blocker	Dose (mg)	Doses, *N*	Reported outcome of DRESS	Base treatment	Ref
4	f	54	DRESS	7	Esomeprazole and piperacillin suspected	> 4	Benralizumab	30	1	Clinical improvement	Methylprednisolone	22
5	f	58	DRESS	8	Midazolam suspected	> 4	Benralizumab	30	1	Clinical improvement, lethal outcome (COVID-19)	Methylprednisolone	22
6	m	43	DRESS	8	Cefepime suspected	6.7	Benralizumab	30	2	Clinical improvement	Methylprednisolone IVIG	23
7	f	87	DRESS	5	Allopurinol and pregabalin suspected	1.25	Benralizumab	30	1	Clinical improvement	Methylprednisolone	24
8	m	74	DRESS	6	Allopurinol suspected	5.31	Benralizumab	30	1	Clinical improvement	Methylprednisolone	24
9	f	67	DRESS	7	Ibuprofen and paracetamol suspected	19.35	Benralizumab Mepolizumab	30/100 mg	1/2	Clinical improvement, relapse	Methylprednisolone	24
10	f	45	DRESS	n/a	Lamotrigine suspected	n/a	Mepolizumab	300–500 mg	> 4	Clinical improvement	Methylprednisolone Mycophenolate mofetil Cyclosporine	25
11	f	50	DRESS/AGEP overlap	n/a	Ciprofloxacin suspected	1.5	Mepolizumab	300 mg	2	Clinical improvement	Prednisolone Methylprednisolone Cyclophosphamide Cyclosporine	26
12	m	56	DRESS	> 4	Pregabalin suspected	7.1	Mepolizumab	2 × 300mg 1 day apart, then 300/100 mg	> 3	Clinical improvement	Methylpredisolone	27
13	f	56	DRESS	6	Sulfamethoxazole/trimethoprim suspected	6.4	Mepolizumab	100 mg	3	Clinical improvement, relapse	Dexamethasone IVIG	28
14	f	62	DRESS	5	Imatinib (not stopped)	1.69	Reslizumab	100–200 mg	2	Clinical improvement, re-exposure with imatinib, relapse	Dexamethasone	29

*IVIG* intravenous immunoglobulin, *F* female, *M* male, *n/a* not applicable

### Mepolizumab in DRESS

Out of 14 patients (50%) were treated with mepolizumab, including the subject treated with both benralizumab and mepolizumab (cases 1, 2, 9–13) [24–28]. Two of them from our clinic (Table 1): Amoxicillin was identified as the causal drug for DRESS in a 62-year-old man (case 1). Sensitization to amoxicillin was documented later by a lymphocyte transformation test (LTT). Since treatment with systemic high-dose CS did not result in any clinical improvement (hepatitis, renal failure under dialysis), 100 mg mepolizumab was given at day 14. Within 2 days, clinical improvement was noticed with normalization of blood eosinophilia (0.04 G/l). Due to relapses (exanthema, facial swelling, increases of transaminases (2-fold) and increasing eosinophilia (0.44 G/l and 0.75 G/l, respectively)), two additional injections of 100 mg mepolizumab were given at day 37 and 117, resulting in complete remission of DRESS. The other patient, a 70-year-old man, received a single dose of 100 mg mepolizumab because of a toxic epidermal necrolysis (TEN)/acute generalized exanthematous pustulosis (AGE)P/DRESS overlap syndrome (case 2). While peripheral eosinophils declined rapidly, clinical condition improved slowly. As mepolizumab did not have a significant impact on the clinical course, possibly because TEN was the primary pathophysiological mechanism of the reaction, it was stopped.

Mepolizumab had a good therapeutic effect, but similar as in our first patient, it had to be administered more than once in all cases published. In the first article on mepolizumab used in DRESS, initial improvement was achieved with high-dose CS [28]. Clinical course, however, was characterized by multiple severe relapses, even under treatment with CS. A single injection of 100 mg mepolizumab then resulted in normalization of the eosinophils within 3 days and improvement of the skin rash within a week. Nevertheless because of relapses, continuation of CS for 3 months was necessary. In two other reports, relapses were successfully treated with mepolizumab: necrotizing eosinophilic myocarditis was initially treated with 300 and 500 mg mepolizumab [25]. Symptoms were in control despite CS reduction, but treatment had to be continued for almost 1 year. Truong et al. also reported a successful outcome of eosinophilic myocarditis which was treated with 300 mg mepolizumab twice with a 4-week interval in combination with cyclophosphamide and cyclosporine [26]. Another report of a severe DRESS with pulmonary involvement showed a significant improvement within 13 days after administration of 600 mg mepolizumab (two 300 mg doses over 2 consecutive days) [27]. Nonetheless, a longer treatment period with mepolizumab at 4‑week intervals was required. The 7th case is described in the section “Benralizumab” [24].

### Benralizumab in DRESS

To date seven DRESS case reports with patients treated with benralizumab have been published, including the one from our database (Table 1; [22–24]): The suspected trigger in our case was metamizole (case 3). Although CS were introduced, the patient developed persistent, histologically proven eosinophilic hepatitis. Benralizumab was given three times at a dose of 30 mg at monthly intervals. This procedure led to a rapid suppression of eosinophils and the hepatitis improved. CS could be reduced to under 10 mg prednisolone after 7 months with only mild transient elevation of liver transaminases.

Remarkably, in most of the reported cases (4/7) a single dose of benralizumab 30 mg seems to be sufficient for recovery [22, 24]. Schmid-Grendelmeier et al. reported on 2 patients with DRESS and concomitant severe coronavirus disease 2019 (COVID-19) infection requiring intensive care treatment [22]. In both cases, rapid decrease of peripheral eosinophils within 2 days was noticed together with clinical improvement of skin and liver inflammation. One subject died because of COVID-19 complications. The same group published 3 additional case reports on DRESS [24]. Two of them revealed a rapid and sustained improvement after a single dose of 30 mg benralizumab. The third subject had a relapse 4 months later but improved after a further anti-IL5 treatment with mepolizumab (switched because of lack of benralizumab).

Dose-repetition of benralizumab was also necessary in the publication by Mesli et al. to safely taper CS [23]. Severe hemophagocytic lymphohistiocytosis with increasing eosinophilia despite CS led to the administration of 30 mg benralizumab, resulting in rapid improvement of lymphohistiocytosis, but also rash and other organ manifestations within 16 days. To be able to reduce the systemic steroids, another injection was necessary after 4 weeks.

### Reslizumab in DRESS

In the current literature, only one case has been published [29]. Interestingly, the authors described that the suspected causing drug, imatinib, could be reintroduced under the treatment of IL‑5 blockade. Whether or not imatinib was the culprit drug remains unclear since it was not tested according to the recommendations for drug allergy. Re-exposures with imatinib led twice to DRESS relapses. The administration of reslizumab at doses of 100 mg resulted in a rapid normalization of peripheral eosinophilia and improvement of clinical symptoms. After 2 weeks, because of a mild rash, reslizumab at a dose of 200 mg was administered, followed by complete recovery. Subsequently the patient tolerated imatinib for at least 2 more years.

## Discussion

Although IL‑5 axis targeted treatments are used nowadays in numerous TH-2-mediated diseases, there are few reports concerning their use in eosinophilic complications such as drug hypersensitivity reactions. To date, 11 case reports have been published, with 3 more cases from our database [22–29]. Currently, IL-5/IL-5R blockade is mainly used in refractory DRESS, in severe DRESS relapses, and in cases with severe organ manifestation. In the majority of cases (13/14, 93%), rapid improvement with good suppression of peripheral eosinophilia and consecutive reduction of CS could be achieved. Schmid-Grendelmeier et al. showed that treatment with benralizumab not only decreased IL‑4 and IL‑5 levels, but also had an influence on proteins, related to cytotoxic T‑cell function [22]. Several months of treatment are probably necessary, especially when IL‑5 antagonists such as mepolizumab and reslizumab are used. In two cases of our database as well as in published cases, relapses could be documented a few weeks after mepolizumab administration [27, 28].

In brief, 2–3 injections of mepolizumab or reslizumab at monthly intervals are necessary for complete remission even at doses up to 600 mg. On the contrary benralizumab once at a dose of 30 mg was sufficient in most patients. Due to the mode of action with depletion of eosinophils, it is expected that fewer injections are necessary with benralizumab. Although it seems that benralizumab has a more pronounced effect on DRESS, the choice of which drug to use is probably based on availability, as it is an off-label treatment. The small number of cases does not allow a reliable statement which of the three drugs is comparatively more effective.

An interesting observation is the one case with DRESS who tolerated imatinib, the putative DRESS-causing drug, for at least 2 years after treatment with reslizumab, even after discontinuation of IL‑5 blockade [29]. Unfortunately, no workup was performed to confirm imatinib as the causative drug.

The selection of patients to treat with IL-5/IL-5R blockade is of great importance. In our collective, the patient with the overlap of TEN, AGEP, and DRESS showed little clinical improvement, which may indicate that the underlying DHR of TEN/AGEP was probably stronger than that of DRESS, and IL‑5 was not the primary cytokine involved (case 2).

Our analysis of 14 DRESS cases suggests that by blocking the IL-5/IL-5R pathway, rapid improvement can be achieved in most severe DRESS cases. Within 2 days to 2 weeks, a rapid decrease in peripheral eosinophils is observed, accompanied by clinical improvement in most patients. The therapeutic effects of CS or immunosuppressants, which are usually administered before or concomitantly with the administration of an IL-5-blocking drug, must be considered when evaluating therapeutic success in the clinical course. The potential benefit of IL-5/IL-5R blockade is the targeted reduction in dose or duration of CS treatment, thereby minimizing CS-induced complications and disease burden. Furthermore, IL-5/IL-5R blockade seems to be effective not only in the initial DRESS course, but also in DRESS relapses. Disadvantages of IL-5/IL-5R blockade are the high cost of treatment, regardless of the biologic used, and potential side effects such as headache, nasopharyngitis, and respiratory tract infections. On the other hand, using it for a few months can shorten hospitalization and avoid DRESS-associated complications, which in turn can prevent subsequent costs.

Despite clinical improvement in most severe DRESS cases, some questions remain unanswered because of the small number of DRESS reports with treatment of IL‑5 blocking drugs. Whether early blockade of the IL‑5 axis can truly prevent or at least reduce late complications such as immune dysregulation, multiple drug hypersensitivity syndrome, or persistent organ damage cannot yet be answered. It is also uncertain whether IL-5/IL5R blockade is useful in preventing severe DRESS or could be used directly—in addition to CS—for moderate or mild forms of DRESS. Currently, treatment with mepolizumab, benralizumab, and reslizumab will mostly remain a potential treatment for severe DRESS courses [30]. Based on the reported cases, we propose a use of an IL5/IL-5R blockade in the following situations (Table 3):

- Early use in severe DRESS courses with need of intensive care medicine treatment
- Lack of clinical improvement under high dose of CS (≥ 1 mg/kg prednisone equivalent per day, treatment for > 1 week) and persistent eosinophilia ≥ 1.0 G/l
- Additional use of an immunosuppressive drug needed despite high dose of CS
- Severe DRESS with concomitant infectious disease
- Evidence of severe/life threatening organ damage (e.g., myocarditis, eosinophilic pneumonia, hepatitis) at the beginning or DRESS relapse

**Table 3:** Recommendation for the use of IL-5/IL-5R antagonists in drug reaction with eosinophilia and systemic symptoms

*When to use IL-5/IL-5R blockade?*
– Severe DRESS course with need of intensive care medicine
– No clinical improvement under high-dose systemic steroids (≥ 1 mg/kg prednisone equivalent per day) for ≥ 7 days and persistent eosinophilia above 1.0 G/l
– Additional use of an immunosuppressive drug (e.g., cyclosporine) needed despite high-dose CS
– Severe DRESS with concomitant infectious disease
– Evidence of severe/life threatening organ damage (e.g., myocarditis, eosinophilic pneumonia, hepatitis) at the beginning or at a DRESS relapse
*Duration of treatment*
– Recommendation of 3 months at 4‑week intervals for mepolizumab and reslizumab
– A single dose of benralizumab might be sufficient
*Treatment dose recommendation*
– Mepolizumab: 300 mg
– Benralizumab: 30 mg
– Reslizumab: according to body weight

For treatment duration, we recommend a period of at least 3 months, especially if mepolizumab or reslizumab is used with injections at 4‑week intervals at a dose of 300 mg or according to the body weight. For benralizumab, a single dose at 30 mg might be sufficient.

The limitation of our study is the small case number and the lack of comparability in a control group. Therefore, prospective studies on the treatment of DRESS are urgently needed.

## Conclusion

Patients with DRESS often require longer-term immunosuppressive treatment, especially with high dose CS. Blockade of the IL-5/IL-5R axis appears to be a promising treatment, at least in severe DRESS, which may allow more rapid reduction of CS and shortening of hospital stay. Nevertheless, it is usually necessary to monitor the therapy and clinical course over several months.
